# Accuracy of the qSOFA Score and RED Sign in Predicting Critical Care Requirements in Patients with Suspected Infection in the Emergency Department: A Retrospective Observational Study

**DOI:** 10.3390/medicina56010042

**Published:** 2020-01-19

**Authors:** Jong Eun Park, Sung Yeon Hwang, Ik Joon Jo, Min Seob Sim, Won Chul Cha, Hee Yoon, Tae Rim Kim, Gun Tak Lee, Hye Seung Kim, InSuk Sohn, Tae Gun Shin

**Affiliations:** 1 Department of Emergency Medicine, Samsung Medical Center, Sungkyunkwan University School of Medicine, Seoul 06351, Korea; jongeun7.park@samsung.com (J.E.P.); gerup@hanmail.net (S.Y.H.); drjij@skku.edu (I.J.J.); minsub01.sim@samsung.com (M.S.S.); wc.cha@samsung.com (W.C.C.); wildhi.yoon@samsung.com (H.Y.); taerimi.kim@samsung.com (T.R.K.); guntaklee@samsung.com (G.T.L.); 2 Statistics and Data Center, Samsung Medical Center, Sungkyunkwan University School of Medicine, Seoul 06351, Korea; hyeseung.kim@sbri.co.kr (H.S.K.); insuk.sohn@samsung.com (I.S.)

**Keywords:** infection, mortality, sepsis, qSOFA, RED sign

## Abstract

*Background and objectives*: We aimed to compare the accuracy of positive quick sequential organ failure assessment (qSOFA) scores and the RED sign in predicting critical care requirements (CCRs) in patients with suspected infection who presented to the emergency department (ED). *Materials and Methods*: In this retrospective observational study, we examined adult patients with suspected infection in the ED from June 2018 to September 2018. A positive qSOFA (qSOFA+) was defined as the presence of ≥2 of the following criteria: altered mental status (AMS), systolic blood pressure (SBP) < 100 mmHg, and respiratory rate (RR) ≥ 22 breaths/min. A positive RED sign (RED sign+) was defined as the presence of at least one of the RED sign criteria: AMS, skin mottling, SBP < 90 mmHg, heart rate >130 beats/min, or RR > 30 breaths/min. A qSOFA/RED+ was defined as the presence of qSOFA+ or RED+. We applied these tools twice using the initial values upon ED arrival and all values within 2 h after ED arrival. The accuracy of qSOFA+, RED+, and qSOFA/RED+ in predicting CCR was assessed. *Results*: Data from 5353 patients with suspected infection were analyzed. The area under the receiver operating characteristic curve (AUC) of RED+ (0.67, 95% confidence interval [CI]: 0.65–0.70) and that of qSOFA/RED+ (0.68, 95% CI: 0.66–0.70, *p* < 0.01) were higher than the AUC of qSOFA+ (0.59, 95% CI: 0.57–0.60) in predicting CCR on ED arrival. The qSOFA/RED+ within 2 h showed the highest accuracy (AUC 0.72, 95% CI: 0.70–0.75, *p* < 0.001). *Conclusions*: The accuracy of the RED sign in predicting CCR in patients with suspected infection who presented at ED was better than that of qSOFA. The combined use of the RED sign and qSOFA (positive qSOFA or RED sign) showed the highest accuracy.

## 1. Introduction

Sepsis is a major health problem affecting millions of people each year and accounts for high mortality among hospitalized patients worldwide [1]. A recent meta-analysis reported a global estimate of 31.5 million cases of sepsis with potentially 5.3 million deaths annually [2]. The overall hospital mortality rate in patients with sepsis ranges between 20% and 40%, reaching as high as 50% in patients with septic shock [3,4,5]. Efforts to improve the survival rate of patients with sepsis and septic shock are still needed [6,7]. Identifying patients at a high risk of sepsis and septic shock would be the first step to initiate proper therapeutic strategies [8].

The quick sequential organ failure assessment (qSOFA) is a simple scoring system using three physiological parameters (respiratory rate (RR) ≥ 22 breaths/min, altered mental status (AMS), and systolic blood pressure (SBP) ≤100 mmHg (1 point each; score range of 0–3 points)). It was originally proposed as a screening tool to identify patients with suspected infection outside the intensive care unit (ICU) who are at a high risk for poor outcomes, including hospital mortality, in accordance with the new sepsis-3 definition [8].

However, the predictive accuracy of qSOFA might be limited according to recent studies, particularly in the initial evaluation of high-risk patients in the emergency department (ED) [9,10]. In the original qSOFA study, ED populations were not analyzed separately from the larger study population, and the poor discriminative ability of qSOFA has raised concerns about its role for ED patients requiring early recognition and timely intervention [11,12]. Additionally, an extreme variation in a single physiological parameter (RED sign) is not considered to be positive in the qSOFA system. For example, hypotension, severe tachypnea or tachycardia, altered mentation, and skin mottling are signs of critically ill patients with sepsis, and patients with these conditions can be easily examined at their bedside and should therefore not be overlooked. Finally, qSOFA focused on the in-hospital mortality and ICU length of stay as outcomes. For initial evaluation in ED, however, screening patients requiring early critical interventions such as vasopressor use, mechanical ventilator use (MV), renal replacement therapy (RRT), or ICU admission may be more appropriate than predicting in-hospital mortality or ICU stay [13].

This study aimed to compare the accuracy of positive qSOFA and the RED sign in predicting critical care requirements (CCRs) in patients with suspected infection who presented to the ED. We also aimed to evaluate whether the predictive accuracy of qSOFA improved when combined with the RED sign.

## 2. Materials and Methods

### 2.1. Study Design and Setting

This was a single-center, retrospective, observational study in patients with suspected infection who presented to the ED at Samsung Medical Center (a 1960-bed, university-affiliated, tertiary care referral hospital located in a metropolitan city with an annual census over 70,000). The study period was from June 2018 through September 2018. This study was approved on 12/14/2018 by the institutional review board of Samsung Medical Center (IRB number, 2018-12-053); informed consent was waived because this study was retrospective and observational in nature, and patients’ data were anonymized. This study followed the Standards for Reporting of Diagnostic Accuracy Studies guidelines [14].

### 2.2. Study Population

Patients aged 18 years or older and who had suspected infection upon arrival to the ED were included in the study. Patients who were younger than 18 years, had limitations on invasive care (e.g., patients who had previously signed a do-not-resuscitate order), were transferred to another hospital, or had inadequate data on electronic medical records (EMR) were excluded.

### 2.3. Definition and Data Collection

Suspected infection was defined as cases where body fluid culture and antibiotic therapy were performed. The qSOFA score consists of the following three clinical variables: SBP ≤ 100 mmHg, RR ≥ 22 breaths/min, and AMS. The presence of two or more of these criteria was defined as a positive qSOFA score (qSOFA+). The RED sign consists of the following five clinical variables: SBP < 90 mmHg, HR > 130 beats/min, RR > 30 breaths/min, presence of skin mottling on the abdomen or knee, and AMS. The presence of at least one of the RED sign criteria was defined as a positive RED sign (RED+). A positive qSOFA or RED sign (qSOFA/RED+) was defined as the presence of qSOFA+ or RED+. AMS was defined as a Glasgow Coma Scale score of <15 or an alert, voice, pain, and unresponsive scale rating other than “alert” that was documented as a new-onset change from baseline mental status. Any deterioration from baseline mental status in patients who were not fully alert was also regarded as AMS.

Eligible cases were electronically identified based on the definition of suspected infection. The following data were extracted from the hospital database: age, sex, comorbidities, vital signs, mental status, presence of skin mottling, suspected infection focus, initial laboratory tests, survival data, and therapeutic interventions including vasopressor use, MV use, RRT, and ICU admission.

Data regarding AMS and skin mottling were extracted from the initial ED physician’s EMR using a predefined template, and then, initial values were used to quantify these variables. We determined the qSOFA score and the RED sign twice for each patient. The qSOFA or RED sign was first evaluated on ED arrival according to the first vital signs and assessment data. The qSOFA or RED sign within 2 h was considered positive if the criteria were met based on the initial or follow-up values within 2 h after ED arrival. We used the simultaneous values instead of the worst ones for the qSOFA score.

### 2.4. Outcome Measures

The primary outcome was CCRs, which is a composite of vasopressor use, MV use, RRT, and ICU admission. The secondary outcomes were in-hospital and 48-hour mortality after ED arrival.

### 2.5. Statistical Analysis

Standard descriptive statistics were analyzed for all variables. The results are expressed as medians with interquartile ranges (IQR) for continuous variables and as the number of patients with percentages for categorical data. Continuous variables were analyzed using Wilcoxon rank-sum tests, while categorical variables were analyzed using chi-square tests. The prognostic performance of qSOFA+, RED+, and qSOFA/RED+ for outcomes was assessed as sensitivity, specificity, positive predictive value (PPV), negative predictive value (NPV), accuracy, area under the receiver operating curve (AUC), and their corresponding 95% confidence interval (CI). For a comparison of the AUC of two diagnostic modalities, we used a nonparametric approach for dependent receiver operating characteristic curves [15]. Prognostic parameters such as positive and negative predictive values were also calculated using the Wilson’s method and compared using McNemar’s test and Bennett’s test. A *p*-value < 0.05 was considered significant and was corrected using Bonferroni’s method for multiple testing. STATA (version 13.0; StataCorp., College Station, TX, USA) was used for statistical analysis.

## 3. Results

### 3.1. Baseline Characteristics

**Figure 1:**
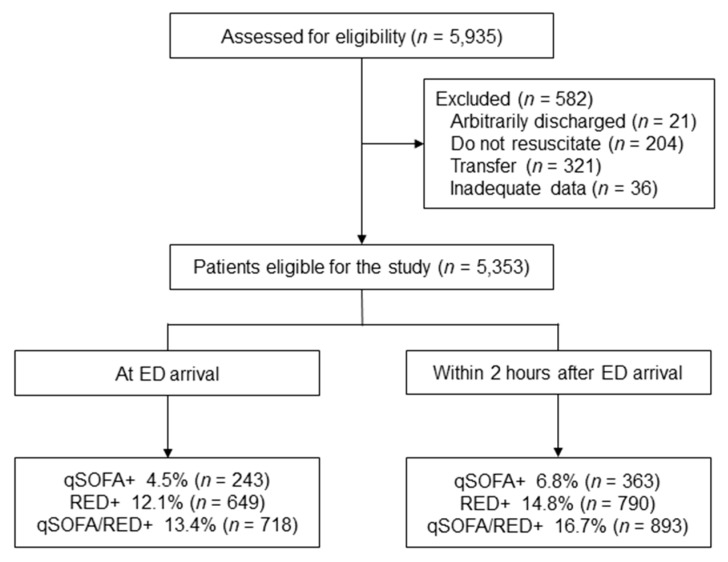
Flowchart of the study process. ED, emergency department; qSOFA+, positive quick sequential organ failure assessment; RED+, positive RED sign; qSOFA/RED+, qSOFA+ or RED+.

A total of 5353 patients with suspected infection who presented to the ED were included (Figure 1), of whom 495 (9.3%) received critical care interventions as the primary outcome. The baseline characteristics of all patients and a comparison between patients who received critical care and those who did not require critical care are shown in Table 1. The median age was 58 years (IQR 47–71), and 51.3% (*n* = 2745) were men. The most common cause of infection was intra-abdominal infection (36.8%), followed by respiratory infection (25.5%). In-hospital mortality was 3.3% (*n* = 179), while the 48-h mortality was 0.7% (*n* = 35).

The proportions of patients with qSOFA+, RED+, and qSOFA/RED+ were 4.5%, 12.1%, and 13.4%, respectively, upon ED arrival, and 6.8%, 14.8%, and 16.7%, respectively, within 2 h. All three indexes were more frequently observed in the critical care group than in the non-critical care group (At ED arrival: qSOFA+ 20.0% vs. 3.0%, RED+ 43.4% vs. 8.9%, qSOFA/RED + 45.9% vs. 10.1%; within 2 h after ED arrival: qSOFA+ 31.7% vs. 4.2%, RED+ 53.7% vs. 10.8%, qSOFA/RED+ 57.4% vs. 12.5%, respectively).

### 3.2. Outcomes

The primary and secondary outcomes are presented in Table 2.

**Figure 2:**
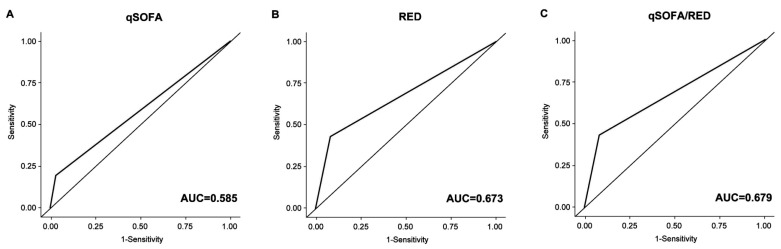
Receiver operating characteristic curve for predicting critical care requirements on ED arrival. (**A**) qSOFA+, (**B**) RED+, and (**C**) qSOFA/RED+. qSOFA+, positive quick sequential organ failure assessment; RED+, positive RED sign; qSOFA/RED+, qSOFA+ or RED+; AUC, area under the receiver operating characteristic; ED, emergency department.

**Figure 3:**
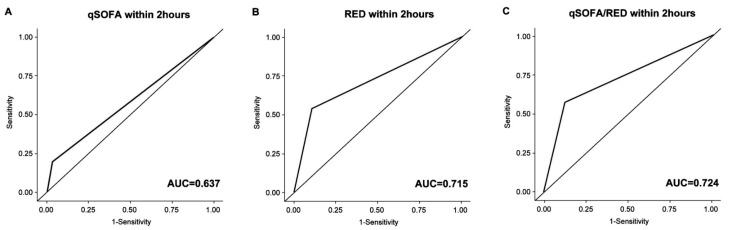
Receiver operating characteristic curve for predicting critical care requirements within 2 h after ED arrival. (**A**) qSOFA+, (**B**) RED+, and (**C**) qSOFA/RED+. qSOFA+, positive quick sequential organ failure assessment; RED+, positive RED sign; qSOFA/RED+, qSOFA+ or RED+; AUC, area under the receiver operating characteristic; ED, emergency department.

The qSOFA/RED+ showed significantly increased sensitivity (46%, 95% CI: 42–50% upon ED arrival; 57%, 95% CI: 53–62% within 2 h) for predicting CCRs compared with qSOFA+ or RED+. The AUC of RED+ upon ED arrival was greater than that of qSOFA+ upon ED arrival (0.673, 95% CI: 0.650–0.695 vs. 0.585, 95% CI: 0.567–0.603, *p* < 0.001 corrected by Bonferroni’s method) for predicting CCRs (Table 3, Figure 2). The AUC of qSOFA/RED+ upon ED arrival was also higher than that of RED+, but it was not statistically significant (*p* = 0.154 corrected by Bonferroni’s method). The AUC of RED+ within 2 h for predicting CCRs was also significantly higher than that of qSOFA+ within 2 h (0.715, 95% CI: 0.692–0.737 vs. 0.637, 95% CI: 0.617–0.658, *p* < 0.001 corrected by Bonferroni’s method) (Table 3, Figure 3). The AUC of qSOFA/RED+ within 2 h was higher than that of RED+ within 2 h, but the statistical significance was marginal (0.724, 95% CI: 0.702–0.747 vs. 0.715, 95% CI: 0.692–0.737, *p* = 0.056 corrected by Bonferroni’s method). Receiver operating characteristic curves for predicting 48-h and in-hospital mortality are elsewhere (see Supplementary Materials, Figures S1 and S2).

## 4. Discussion

In this study, we compared the accuracy of positive qSOFA and RED sign in predicting the need for critical care in patients with suspected infection who presented to the ED. RED sign showed better prognostic accuracy for predicting CCRs than qSOFA. It also showed better performance in predicting in-hospital mortality and 48-h mortality than qSOFA. The combination of qSOFA and RED sign was the most accurate model for predicting outcomes.

The original qSOFA study was conducted among patients from outside the ICU, and it revealed that the rate of patients with severe infection was relatively low and that ED populations were not analyzed separately [8]. Several studies investigated the role of qSOFA as a screening tool in the ED. Moskowitz et al. [13] compared qSOFA and systemic inflammatory response syndrome criteria in predicting the need for critical care intervention (vasopressor use, assisted ventilation, continuous insulin infusion, ≥4000 mL intravenous fluid within 12 h of ICU admission, placement of invasive catheters, or RRT) in patients with suspected infection who presented to the ED. They showed that the sensitivity of qSOFA+ was only 13% in predicting the need for critical care intervention. Among patients with a qSOFA of 1, 23.5% received a critical care intervention. Williams et al. [12] demonstrated that qSOFA+ had high specificity but poor sensitivity (96.1%; 95% CI, 95.7–96.6% and 29.9%; 95% CI, 27.9–31.8%, respectively), with an AUC of 0.73 (95% CI, 0.72–0.74) for organ dysfunction. Henning et al. [16] performed secondary analysis of three prospectively collected observational cohorts of ED patients with infection. They showed that qSOFA+ had a sensitivity of 52% (95% CI 46–57%) and specificity of 86% (95% CI 85–87%) in predicting mortality. One study showed relatively high sensitivity (70%, 95% CI 59–80) in predicting in-hospital mortality; however, the worst value during the ED stay was used to calculate the qSOFA score in the study [17]. Our study was consistent with previous studies and suggests that qSOFA alone has limitations for evaluating patients with suspected infection in the ED. Therefore, qSOFA should be interpreted with caution [18,19].

Various studies have been conducted to improve the performance of qSOFA. Shetty et al. [20] performed a retrospective cohort study of suspected or proven sepsis patients presenting to ED. They added lactate level (≥2 mmol/L) to the qSOFA score of ≥2 and showed improved performance compared to qSOFA of ≥2 alone for identifying patients at a risk for in-hospital mortality or prolonged ICU stay (AUC 0.74 [95% CI 0.73–0.74] vs. 0.68 [95% CI 0.68–0.69]; sensitivity 65.5% [95% CI 62.6–68.4] vs. 47.6% [95% CI 44.6–50.6]). We used an extreme physiologic parameter or physical finding, which is not considered as positive in the qSOFA scoring system. The RED sign showed better performance than qSOFA in predicting CCRs in our study and can be easily detected at the bedside. RED signs should be a trigger for rapid intervention in patients with suspected or confirmed infection. Considering the limitations of qSOFA and our results, we suggest that a reasonable and practical approach may be to screen for a RED sign at the initial evaluation and check the qSOFA and RED signs serially if there is no abnormal finding.

There are several strengths to our study. First, our study focused on the composite outcome of CCRs, which is an important issue for ED physicians in starting early aggressive management for sepsis. Second, we extracted objective data from EMRs of a large patient population. Thus, we could include the majority of adult patients who had suspected infection regardless of disease severity, and very few patients were excluded from the study. Therefore, our study could show rigorous statistics and significant findings. Third, we investigated qSOFA and RED sign using the initial and follow-up data from the early period. We did not use the worst value during the entire ED stay because its clinical application at the bedside could be limited.

This study also had some limitations. First, this was a single-center study conducted in the ED of a tertiary academic institution. The incidence rates of outcomes may be different from those of other settings. Hence, the generalizability of these results to other settings is limited, and external validation is required. Second, this study had inherent limitations as a retrospective observational study. Most of the data were electronically extracted from the hospital database. Notably, unlike vital signs that were frequently reassessed, data including mental status and skin mottling might be relatively lacking. However, adding initial assessment of these variables and recording it on the EMR was mandatory in our institution. In the original qSOFA study, over 75% of patients outside of the ICU in the derivation cohort lacked GCS data. Third, the study period was brief. However, our institution, as a tertiary referral hospital located in an urban area, had a large volume of patients.

## 5. Conclusions

Our study revealed that the accuracy of RED sign in predicting CCRs and mortality in patients with suspected infection who presented at ED was better than that of qSOFA. The combined use of RED sign and qSOFA (positive qSOFA or RED sign) showed the highest diagnostic accuracy, and it can be used as a more accurate screening tool for identifying high-risk patients who will need critical care intervention compared to the qSOFA or RED sign alone.

## Figures and Tables

**Table 1:** Baseline characteristics of patients.

	Total (*n* = 5353)	Critical Care (*n* = 495)	No-Critical Care (*n* = 4858)	*p*-Value
Age (year)	58 (47–71)	64 (55–75)	60 (46–71)	<0.001
Male sex	2745 (51.3)	287 (58.0)	2458 (50.6)	0.002
Infection focus				
Lung	1365 (25.5)	163 (32.9)	1202 (24.7)	<0.001
Abdomen	1969 (36.8)	179 (36.2)	1790 (36.9)	0.763
Urinary tract	743 (13.9)	65 (13.1)	678 (13.9)	0.613
Bone/soft tissue	480 (9)	19 (4)	461 (9.5)	<0.001
Neutropenic fever	438 (8.1)	34 (6.9)	404 (8.3)	0.263
Others	261 (4.9)	39 (7.8)	222 (4.5)	0.001
Unknown	179 (3.3)	9 (1.8)	170 (3.5)	0.048
Laboratory test				
Lactate (mmol/L)	1.5 (1.1–2.1)	2.3 (1.6–4)	1.4 (1.1–1.9)	<0.001
CRP (mg/dL)	4.1 (1.1–9.8)	7.9 (2.4–18.3)	3.8 (1–9.1)	<0.001
Vital sign, upon ED arrival				
SBP	120 (104–137)	110 (89–133)	120 (105–137)	<0.001
HR	97 (83–110)	106 (88–123)	96 (83–109)	<0.001
RR	18 (18–20)	20 (18–24)	18 (18–20)	<0.001
Vital sign, 2 h after ED arrival				
SBP	115 (103–131)	104 (91–125)	116 (104–132)	<0.001
HR	90 (78–102)	99 (85–116)	89 (78–101)	<0.001
RR	18 (16–20)	20 (18–24)	18 (16–20)	<0.001
qSOFA+ upon ED arrival	243 (4.5)	99 (20.0)	144 (3.0)	<0.001
qSOFA+ within 2 h after ED arrival	363 (6.8)	157 (31.7)	206 (4.2)	<0.001
*RED+ upon ED arrival	649 (12.1)	215 (43.4)	434 (8.9)	<0.001
SBP < 90 mmHg	207 (2.4)	119 (26.8)	87 (4.0)	<0.001
HR > 130 beats/min	298 (3.2)	69 (15.9)	116 (5.3)	<0.001
RR > 30 breaths/min	90 (1.0)	18 (4.2)	21 (1.0)	<0.001
Skin mottling	10 (0.2)	7 (1.4)	3 (0.1)	<0.001
Altered mental status	80 (1.5)	36 (7.3)	44 (0.9)	<0.001
RED+ within 2 h after ED arrival	790 (14.8)	266 (53.7)	524 (10.8)	<0.001
SBP < 90 mmHg	197 (2.1)	102 (23)	44 (2.0)	<0.001
HR > 130 beats/min	83 (0.9)	50 (11.3)	33 (1.5)	<0.001
RR > 30 breaths/min	64 (0.7)	30 (6.8)	34 (1.6)	<0.001
qSOFA/RED+ upon ED arrival	718 (13.4)	227 (45.9)	491 (10.1)	<0.001
qSOFA/RED+ within 2 h after ED arrival	893 (16.7)	284 (57.4)	609 (12.5)	<0.001
48-h mortality	35 (0.65)	24 (4.9)	11 (0.2)	<0.001
In-hospital mortality	177 (3.3)	83 (16.8)	94 (1.9)	<0.001

The data are presented as mean ± standard deviations, median (interquartile ranges), or numbers (%). CRP, C-reactive protein; ED, emergency department; SBP, systolic blood pressure; HR, heart rate; RR, respiratory rate; qSOFA+, positive quick sequential organ failure assessment; RED+, positive RED sign; qSOFA/RED+, positive qSOFA or RED sign. * The presence of at least one of the RED sign criteria (SBP < 90 mmHg, HR > 130 beats/min, RR > 30 breaths/min, presence of skin mottling and altered mental status) was defined as positive RED sign.

**Table 2:** Primary and secondary outcomes.

Outcomes	Total	On ED Arrival			Within 2 h after ED Arrival		
		qSOFA+ (*n* = 243)	* RED+ (*n* = 649)	qSOFA/RED+ (*n* = 718)	qSOFA+ (*n* = 363)	RED+ (*n* = 790)	qSOFA/RED+ (*n* = 893)
Critical care requirements ^†^	495 (9.3)	99 (40.7)	215 (33.1)	227 (31.6)	157 (43.3)	266 (33.7)	284 (31.8)
Vasopressor use	426 (8)	93 (38.3)	206 (31.7)	216 (30.1)	149 (41.0)	253 (32.0)	268 (30.0)
MV use ^‡^	144 (2.7)	34 (14.0)	64 (9.9)	67 (9.3)	46 (12.7)	77 (9.7)	82 (9.2)
RRT ^§^	27 (0.5)	10 (4.1)	15 (2.3)	15 (2.1)	12 (3.3)	19 (2.4)	20 (2.2)
ICU admission	258 (4.8)	55 (22.6)	106 (16.3)	113 (15.7)	86 (23.7)	133 (16.8)	143 (16.0)
48-h mortality	35 (0.65)	14 (5.7)	19 (2.9)	20 (2.8)	20 (5.5)	24 (3.0)	24 (2.7)
In-hospital mortality	177 (3.3)	37 (15.2)	68 (10.5)	78 (10.9)	53 (14.6)	83 (10.5)	93 (10.4)

* The RED sign consists of the following five clinical variables: systolic blood pressure < 90 mmHg, heart rate > 130 beats/min, respiratory rate > 30 breaths/min, presence of skin mottling on the abdomen or knee, and altered mental status. The presence of at least one of the RED sign criteria was defined as a positive RED sign. ^†^ A composite of vasopressor use, MV use, RRT, or ICU admission. ^‡^ Use of MV within 24 h after ED arrival. ^§^ RRT within 24 h after ED arrival. ED, emergency department; qSOFA+, positive quick sequential organ failure assessment; RED+, positive RED sign; qSOFA/RED+, qSOFA+ or RED+; ICU, intensive care unit; MV, mechanical ventilator; RRT, renal replacement therapy.

**Table 3:** Prognostic performance of scoring systems for predicting critical care requirements and mortality in patients with suspected infection.

		Sensitivity, % (95% CI)	Specificity, % (95% CI)	PPV, % (95% CI)		NPV, % (95% CI)	Accuracy, % (95% CI)	AUC (95% CI)
**Requirement of Critical Care**								
On ED arrival	qSOFA+	20 (17–40)	97 (97–97)	41 (35–47)		92 (91–93)	90 (89–91)	0.585 (0.567–0.603)
	RED+	43 (39–48) *	91 (90–92) *	33 (30–37) *		94 (93–95) *	86 (86–88) *	0.673 (0.650–0.695) *
	qSOFA/RED+	46 (42–50) †	90 (89–91) †	32 (28–35) †		94 (94–95) †	86 (84–87) †	0.679 (0.656–0.701)
Within 2 h after ED arrival	qSOFA+	32 (27–36)	96 (95–96)	43 (38–48)		93 (93–94)	90 (89–91)	0.637 (0.617–0.658)
	RED+	54 (50–58) *	89 (88–90) *	34 (30–37) *		95 (94–96) *	98 (85–87) *	0.715 (0.692–0.737) *
	qSOFA/RED+	57 (53–62) †	87 (86–88) †	32 (29–35) †		95 (95–96) †	85 (84–86) †	0.724 (0.702–0.747)
**In–hospital Mortality**								
On ED arrival	qSOFA+	21 (16–27)	96 (95–96)		15 (11–20)	97 (97–98)	94 (93–94)	0.585 (0.555–0.615)
	RED+	38 (32–46) *	89 (88–90) *		10 (8–13) *	98 (97–98) *	97 (86–88) *	0.636 (0.600–0.672) *
	qSOFA/RED+	44 (37–51) †	88 (87–89) †		11 (9–13)	98 (97–98) †	86 (85–87) †	0.659 (0.622–0.696) †
Within 2 h after ED arrival	qSOFA+	30 (24–37)	94 (93–95)		15 (11–19)	98 (97–98)	92 (91–93)	0.620 (0.596–0.654)
	RED+	47 (40–54) *	86 (85–87) *		11 (9–13) *	10 (10–10) *	85 (84–86) *	0.666 (0.629–0.703) *
	qSOFA/RED+	52 (45–60) †	85 (84–86) †		10 (9–13)	98 (98–98) †	83 (82–84) †	0.685 (0.648–0.723)
**48-h Mortality**								
On ED arrival	qSOFA+	40 (25–56)	96 (95–96)	6 (3–9)		100 (99–100)	95 (95–96)	0.679 (0.596–0.761)
	RED+	54 (38–70)	88 (87–89) *	3 (2–5) *		100 (99–100)	88 (87–88) *	0.712 (0.628–0.796)
	qSOFA/RED+	57 (41–72)	87 (86–88) †	3 (2–4)		100 (99–100)	87 (86–88) †	0.720 (0.637–0.803)
Within 2 h after ED arrival	qSOFA+	57 (41–72)	94 (93–94)	6 (4–8)		10 (10–10)	93 (93–94)	0.754 (0.670–0.837)
	RED+	69 (52–81)	86 (84–86) *	3 (2–4) *		10 (10–10) *	85 (84–86) *	0.771 (0.693–0.849) †
	qSOFA/RED+	69 (52–81)	84 (83–85) †	3 (2–4) †		10 (10–10) †	84 (83–85) †	0.761 (0.683–0.839)

* A significant difference was observed between the results of RED+ and those of qSOFA+, *p* < 0.05 corrected by Bonferroni’s method. ^†^ A significant difference was observed between the results of RED+ and those of qSOFA/RED+, *p* < 0.05 corrected by Bonferroni’s method. CI, confidence intervals; PPV, positive predictive value; NPV, negative predictive value; AUC, area under the receiver operating characteristic curve; ED, emergency department; qSOFA+, positive quick sequential organ failure assessment; RED+, positive RED sign; qSOFA/RED+, qSOFA+ or RED+.

## Supplementary Materials

The following are available at online at https://www.mdpi.com/1010-660X/56/1/42/s1. Figure S1: Receiver operating characteristic curve for predicting in-hospital mortality. Figure S2: Receiver operating characteristic curve for predicting 48-h mortality.
