# Supplementary material for: Accuracy of the qSOFA Score and RED Sign in Predicting Critical Care Requirements in Patients with Suspected Infection in the Emergency Department: A Retrospective Observational Study
Source: Medicina (Kaunas). 2020 Jan 19;56(1):42. doi: 10.3390/medicina56010042 (PMC7022561; doi:10.3390/medicina56010042)
Supplement: Supplementary file 1 [file medicina-56-00042-s001.pdf]

SUPPLEMENTARY MATERIALS

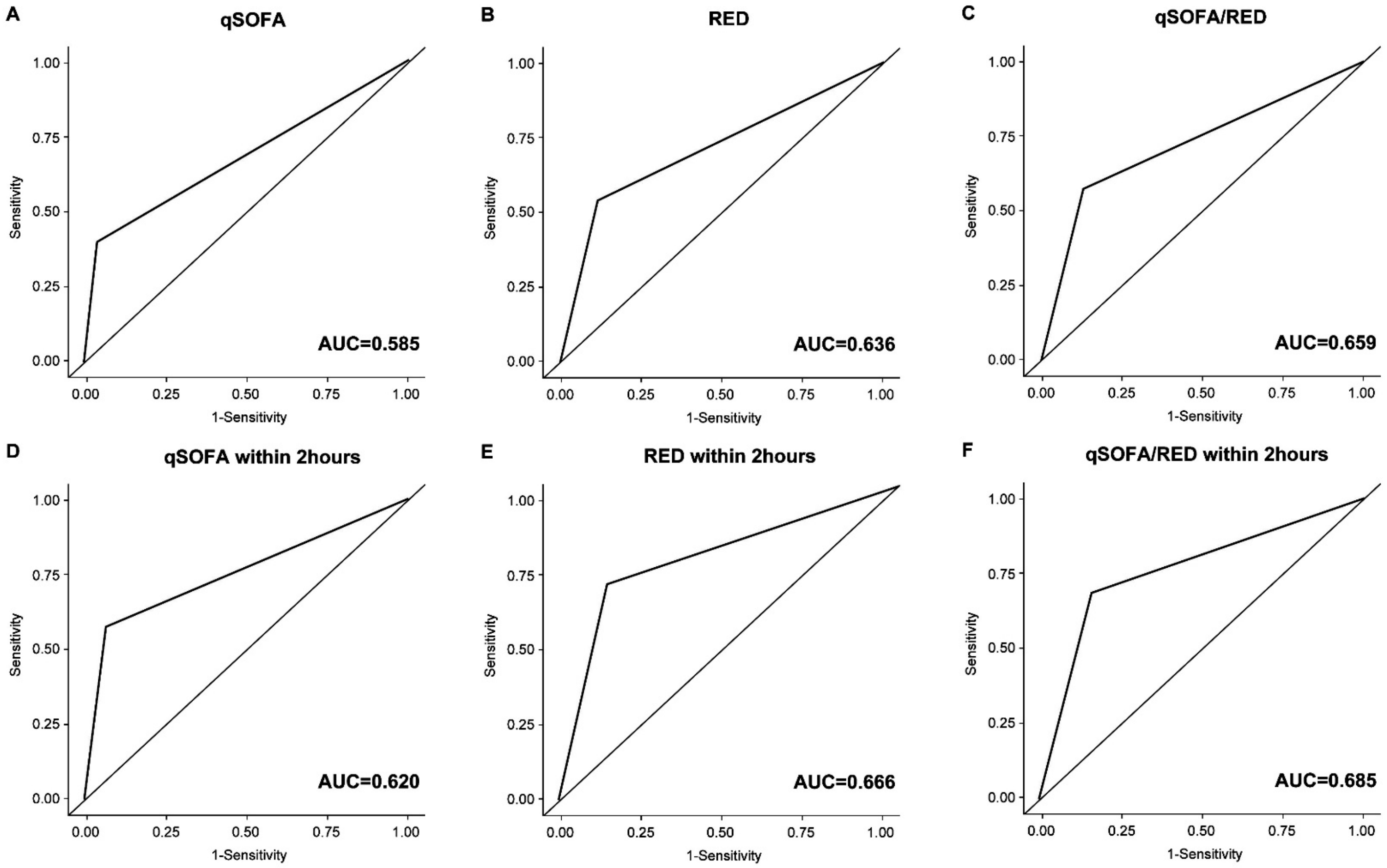

Figure S1. Receiver operating characteristic curve for predicting in-hospital mortality. (A) qSOFA+, (B) RED+, and (C) qSOFA/RED+ on ED arrival; (D) qSOFA+, (E) RED+, and (F) qSOFA/RED+ within 2 hours after ED arrival. qSOFA+, positive quick Sequential Organ Failure Assessment; RED+, positive RED sign; qSOFA/RED+, qSOFA+ or RED+; AUC, area under the receiver operating characteristic; ED, emergency department.

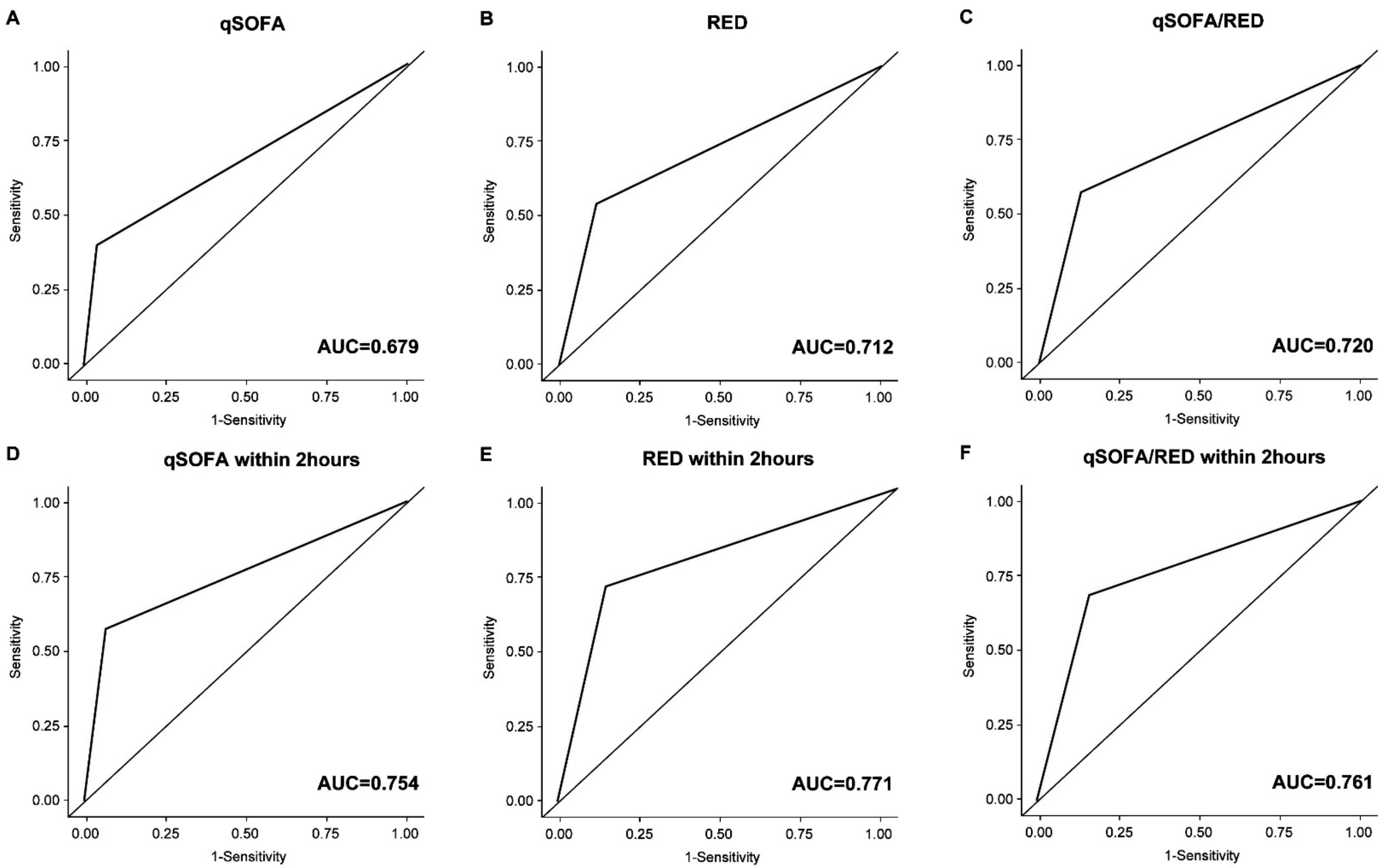

Figure S2. Receiver operating characteristic curve for predicting 48-hour mortality. (A) qSOFA+, (B) RED+, and (C) qSOFA/RED+ on ED arrival; (D) qSOFA+, (E) RED+, and (F) qSOFA/RED+ within 2 hours after ED arrival. qSOFA+, positive quick Sequential Organ Failure Assessment; RED+, positive RED sign; qSOFA/RED+, qSOFA+ or RED+; AUC, area under the receiver operating characteristic; ED, emergency department.
